# Tumour organoids and assembloids: Patient‐derived cancer avatars for immunotherapy

**DOI:** 10.1002/ctm2.1656

**Published:** 2024-04-25

**Authors:** Jie Mei, Xingjian Liu, Hui‐Xiang Tian, Yixuan Chen, Yang Cao, Jun Zeng, Yung‐Chiang Liu, Yaping Chen, Yang Gao, Ji‐Ye Yin, Peng‐Yuan Wang

**Affiliations:** ^1^ Oujiang Laboratory; Key Laboratory of Alzheimer's Disease of Zhejiang Province, Institute of Aging Wenzhou Medical University Wenzhou People's Republic of China; ^2^ Department of Clinical Pharmacology, Xiangya Hospital Central South University Changsha People's Republic of China; ^3^ Institute of Clinical Pharmacology, Hunan Key Laboratory of Pharmacogenetics Central South University Changsha People's Republic of China; ^4^ Engineering Research Center of Applied Technology of Pharmacogenomics Ministry of Education Changsha People's Republic of China; ^5^ National Clinical Research Center for Geriatric Disorders, Xiangya Hospital Central South University Changsha People's Republic of China; ^6^ Department of Thoracic Surgery, Xiangya Hospital Central South University Changsha People's Republic of China; ^7^ Hunan Engineering Research Center for Pulmonary Nodules Precise Diagnosis and Treatment, Xiangya Hospital Central South University Changsha People's Republic of China; ^8^ Xiangya Lung Cancer Center, Xiangya Hospital Central South University Changsha People's Republic of China

**Keywords:** organoid, assembloid, cell therapy, immunotherapy, PDTO biobank

## Abstract

**Background:**

Organoid technology is an emerging and rapidly growing field that shows promise in studying organ development and screening therapeutic regimens. Although organoids have been proposed for a decade, concerns exist, including batch‐to‐batch variations, lack of the native microenvironment and clinical applicability.

**Main body:**

The concept of organoids has derived patient‐derived tumour organoids (PDTOs) for personalized drug screening and new drug discovery, mitigating the risks of medication misuse. The greater the similarity between the PDTOs and the primary tumours, the more influential the model will be. Recently, ‘tumour assembloids’ inspired by cell‐coculture technology have attracted attention to complement the current PDTO technology. High‐quality PDTOs must reassemble critical components, including multiple cell types, tumour matrix, paracrine factors, angiogenesis and microorganisms. This review begins with a brief overview of the history of organoids and PDTOs, followed by the current approaches for generating PDTOs and tumour assembloids. Personalized drug screening has been practised; however, it remains unclear whether PDTOs can predict immunotherapies, including immune drugs (e.g. immune checkpoint inhibitors) and immune cells (e.g. tumour‐infiltrating lymphocyte, T cell receptor‐engineered T cell and chimeric antigen receptor‐T cell). PDTOs, as cancer avatars of the patients, can be expanded and stored to form a biobank.

**Conclusion:**

Fundamental research and clinical trials are ongoing, and the intention is to use these models to replace animals. Pre‐clinical immunotherapy screening using PDTOs will be beneficial to cancer patients.

**Key Points:**

- The current PDTO models have not yet constructed key cellular and non‐cellular components.
- PDTOs should be expandable and editable.
- PDTOs are promising preclinical models for immunotherapy unless mature PDTOs can be established.
- PDTO biobanks with consensual standards are urgently needed.

## Graphical abstract

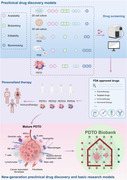

## 1 ORGANOID AND ASSEMBLOID TECHNOLOGY

Organoids have been emerging models in the last decade for studying organ development and prediction of clinical treatment regimens. One of the crucial capabilities in organoid technology is the development of the microstructure and microenvironment of the particular organ.^1^ Given the disparity between two‐dimensional (2D) cell culture and the in vivo microenvironment, the potential uses of organoids as a research model have garnered significant attention in tissue development, disease modelling, clinical diagnosis, drug screening and personalized medicine.^2^

**FIGURE 1:**
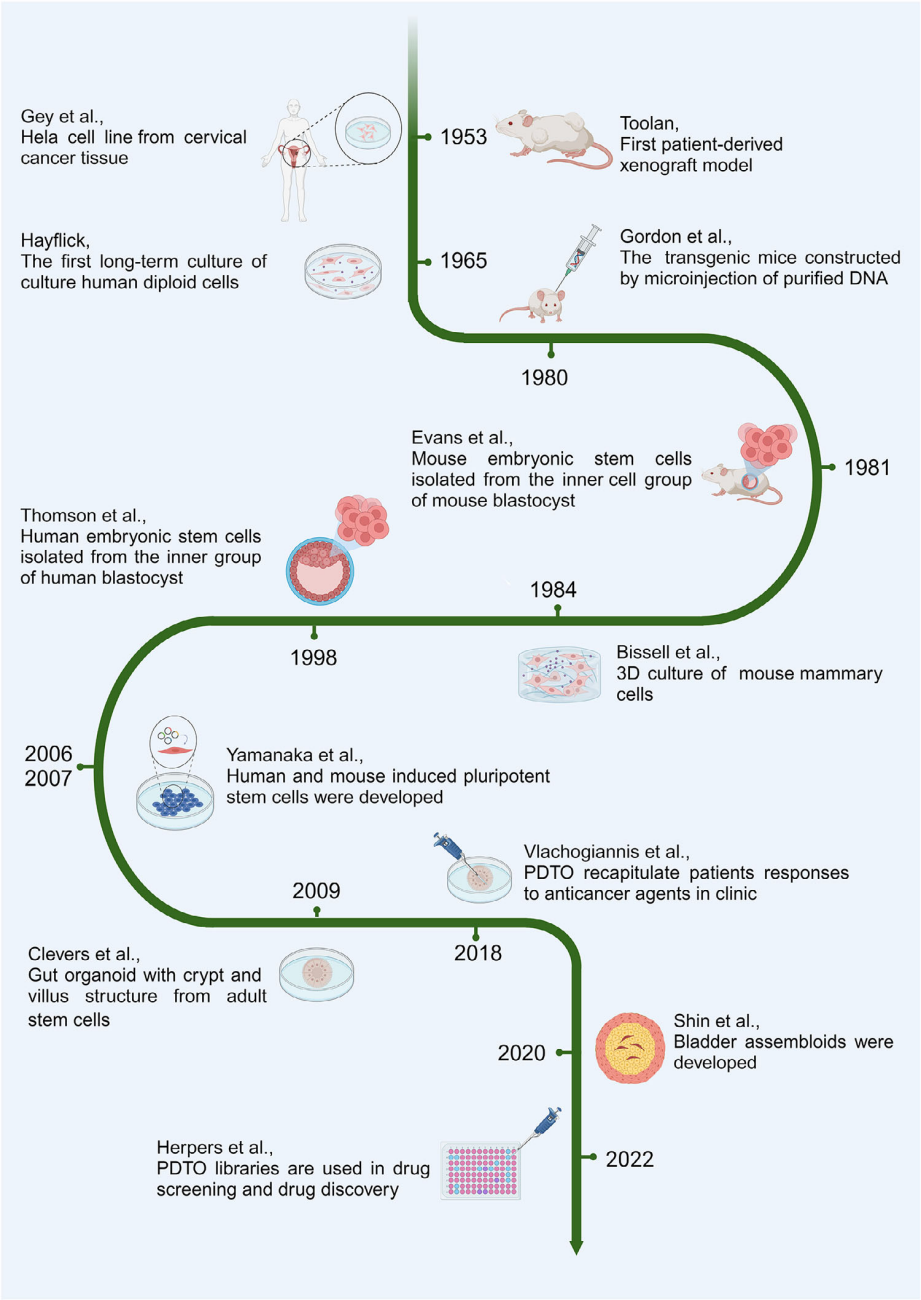
Milestones of the organoid and PDTO development. Cell culture technology has been used for drug discovery for over one century. 2D and 3D models have been generated. Due to the need for an in vivo microenvironment, mouse models have been used. Thanks to organoid technology, the first PDTO was named in 2011. To date, PDTOs have been applied for drug screening practices, but only a few are applicable to predict immunotherapy. In the future, high‐quality PDTOs will be essential pre‐clinical models for immunotherapy.

The advances in the mammalian cell culture,^3^, ^4^ the development of genetically engineered animal models^5^, ^6^ and the gene editing technologies such as CRISPR/Cas9^7^ facilitate the creation of research models. Since the mid‐20th century, researchers have continuously developed various cell culture methods to understand complex cell behaviours and establish a better therapeutic regimen.^8^ Meanwhile, researchers have realized that cell co‐culture technology is vital following the success of blastocyst formation through the co‐culture of human oviduct epithelial cells and embryos.^9^ The cell co‐culture models,^10^ material‐assistant 3D cell culture models^11^ and cancer patient‐derived xenografts (PDX) models^12^ have all provided essential research bases for biomedicine (Figure 1).

Organoids, human organ mimetics, have exponentially developed in the last decade, and they were selected as the ‘Method of the Year 2017’ by *Nature Methods*.^13^ Organoid technology was initially inspired by advanced stem cell technology and has attracted multidisciplinary experts into the field.^14^ The organoids can capture key multicellular and functional features of human organs within micrometres to millimetres.^15^ The first stem cell‐derived organoid was established from Lgr5‐positive mouse intestinal stem cells in 2009.^16^ Afterwards, organoid studies have grown rapidly.^17^ The publications with ‘organoid’ as a keyword have grown approximately 100‐fold in the last decade, from 2009 to 2022 (PubMed website). Over 20 kinds of human organoids have been established, including the stomach,^18^ prostate,^19^ intestines,^20^ liver,^21^ kidneys,^22^ lungs,^23^ brains^24^ and retinal.^25^ Organoid technology complements the shortcomings of 2D cell culture and animal models.^26^ However, most organoids have not yet fully recapitulated the complex human immune microenvironment (IME).^27^, ^28^

Cancer cells are abnormal and threatening cells. Thanks to organoid technology, the first cancer organoid or patient‐derived tumour organoids (PDTOs) was reported from colorectal cancer in 2011.^29^ To date, various cancer organoids, including breast cancer,^30^ bladder cancer,^31^ gastric cancer,^32^ oesophageal adenocarcinoma,^33^ lung cancer^34^ and renal cell carcinoma,^35^ have been generated in the laboratory for fundamental researches. More importantly, cancer organoids using primary cancer cells have been reported to have higher similarity to the original tumours than the immortalized cancer cell lines and PDX models.^36^ This advantage gives cancer organoids an ideal model for cancer research, clinical drug screening and new drug discovery.

Despite the rapid development of PDTOs, the problem remains, such as limited cell types, especially immune cells and capillaries. Thus, a new term named ‘assembloids’ has emerged recently.^37^ Pașca et al. define assembloids as self‐organizing cell systems arising from combinations of different organoids or cell types,^38^ which reflects their conceptual proximity to organoids and implies that they appear to compensate for the limitations of current PDTOs. Assembloids represent a 3D cell co‐culture system, where multiple cell types are mixed simultaneously or sequentially to construct a heterogeneous tissue microenvironment.^39^

Assembloid technology, owing to its enhanced structural organization, has been swiftly integrated into domains, such as tissue remodelling,^40^ tissue spatial genomics^41^, ^42^ and drug delivery assessment,^43^ among others. Similarly, assembloid technology has found applications in normal tissues, including the brain^44^ and the gastrointestinal tract,^40^ as well as cancers, such as bladder cancer^37^ and small‐cell lung cancer.^45^ Many bioengineering tools have been proposed to facilitate assembloid fabrications, such as human brain assembloids using microfluidics,^44^ and spatially constructing glioma organoids using 3D bioprinting.^46^ Although the assembloid approaches differ from the organoid techniques, the ultimate goal is the same for clinical uses. Clinical assessment of drug delivery and efficiency are the leading applications of assembloids. The rapid growth of the assembloid research highlights the current limitations of existing PDTOs and offers an alternative in the field.

The following sections discuss the progress of the current PDTO and tumour assembloids technology, the current applications in personalized drug screening and the future applications in immunotherapy. Finally, insights regarding the future PDTOs and their clinical benefits are proposed.

## 2 PDTOS AND ASSEMBLOIDS

**FIGURE 2:**
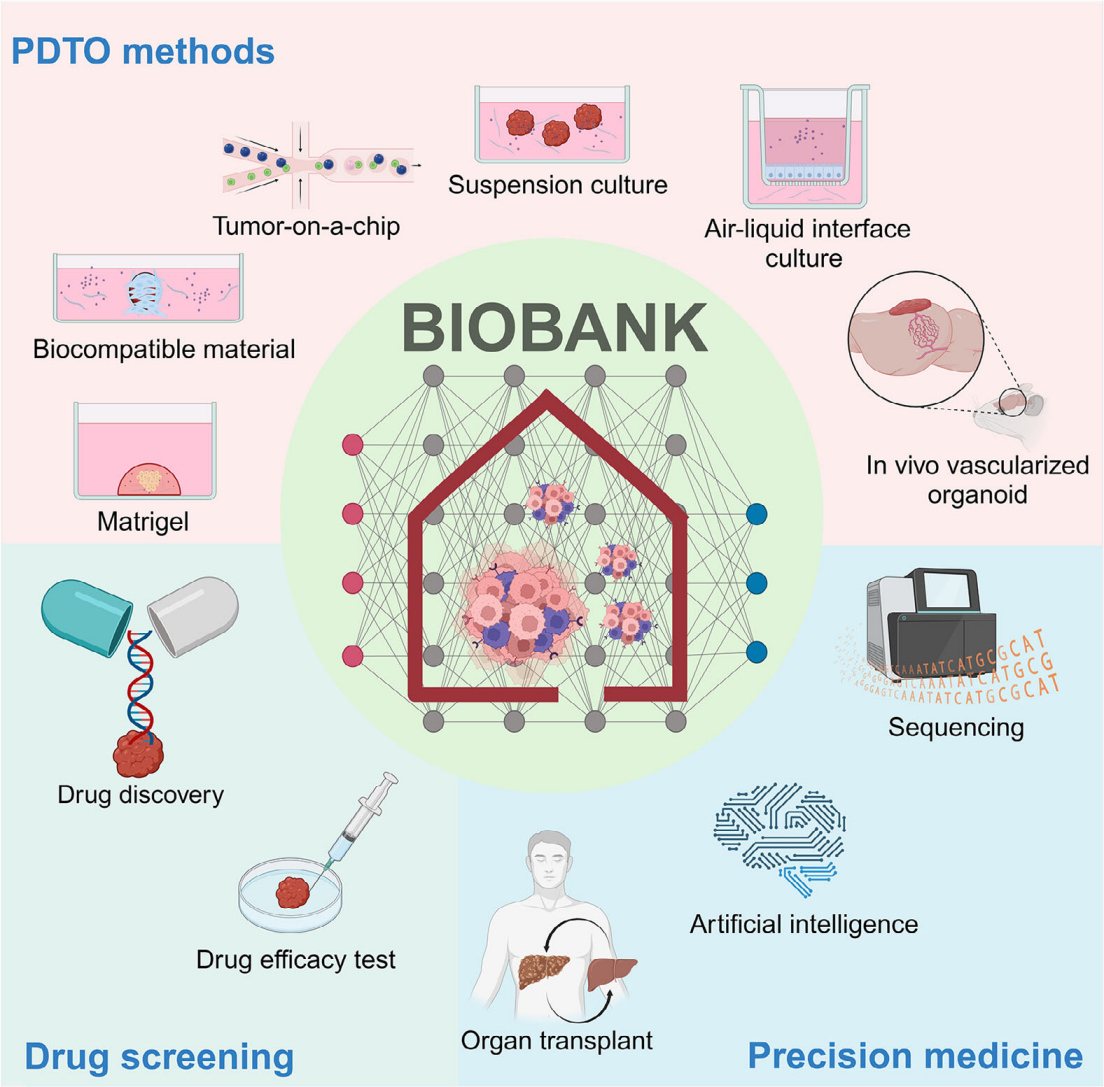
The PDTO technology and biobank for precision medicine. Biomimicking of the tissue microenvironment is critical in PDTO technology. PDTO technology has the potential in establishing a biobank, facilitating new drug discovery, personalized drug screening and disease modelling.

Significant difficulties in clinical research have highlighted the need for pre‐clinical models to improve the prediction efficacy of clinical outcomes. Since 2011, cancer organoids or PDTOs as tumour avatars have shown great potential in studying cancer progression and screening cancer drugs before clinical treatments.^47^, ^48^ These pre‐clinical tests can reduce the risk of inefficient treatments and animal experiments. This section discusses the current approaches for generating PDTOs or tumour assembloids and their applications in clinical drug screening (Figure 2).

### 2.1 Current approaches for PDTOs and tumour assembloids

PDTO technology enables cancer cells to grow in biomimicking conditions faster and more cost‐effectively than the PDX models.^28^, ^49^ While the predominant PDTO cultures use Matrigel to encapsulate tumour cells,^50^ biomaterials scientists have utilized various hydrogels as the tumour matrices, yielding variety of tumour organoids.^51^ Similar to the tissue engineering approach, nature and synthetic hydrogels have been evaluated for PDTO fabrication. For instance, liver PDTOs using alginate‐gelatin hydrogel were constructed, encapsulating patient‐derived liver tumour multicellular clusters, and used for pre‐clinical personalized drug screening.^47^

Recently, researchers have attempted to incorporate more cell types, such as immune cells, into cancer organoids, trying to recreate an IME.^52^ For instance, patient‐derived CD8^+^ T cells and autologous gastric cancer cells were co‐cultured to form PDTOs. This complex system could predict the efficacy of targeted drugs and improve the patient's prognosis.^53^ A more direct approach is to transplant PDTOs into an animal organ. For example, human stem cell‐derived brain organoids have been implanted into the brains of newborn rats. Surprisingly, human brain organoids can grow under such circumstances, integrate with rat neural tissues and regulate rat behaviour.^54^ PDTOs can be expanded in vivo after transplantation, but extensive studies are needed.

PDTOs have also been integrated into microfluidic devices, so‐called tumour‐on‐a‐chip (TuChip), providing a circulatory and flow system.^55^, ^56^ TuChip can be straightforward and mimic tissue−tumour interactions. For example, a microfluidic platform was designed to replicate the biological mass transport near the arterial end of a capillary within the tumour microenvironment (TME). Primary breast tumour organoids were found to remain viable within the device for 20 days and to induce robust sprouting angiogenesis.^57^ Another study focused on kidney organoids, where they generated vascular networks by the flow on a ‘millifluidic chip’. They solved the problem of vascular immaturity in early induced pluripotent stem cells (iPSCs) derivation.^58^ The same protocol can be applied to the PDTOs. Furthermore, the TuChip platforms can reduce variability among PDTO populations by enabling pre‐selection based on size.^59^, ^60^ An automated, high‐throughput microfluidic 3D organoid culture and analysis system was developed, enabling the application of combinatorial and dynamic drug treatments to multiple cultures and allowing for real‐time analysis of PDTOs, which is valuable for pre‐clinical studies.^61^

The air−liquid interface (ALI) model is a unique design that provides a hypoxia condition and mucus‐like environment for in vitro tissue culture.^62^, ^63^ The ALI model indicates a monolayer of cells or tissue slices cultured on a porous membrane, where the medium is underneath and cells are in contact with air. Therefore, the ALI model is ideal for mimicking the respiratory system. For example, Kuo's group constructed over 100 PDTOs using the ALI method to investigate their retention of the original TME. The success rate of initial inoculation with ALI was 73% and the PDTOs were shown to contain multiple immune elements.^64^ The ALI approach still has limitations, such as technical obstacles, success rate and drug attrition due to diffusion‐based drug tests.^62^

Tumour assembloid technology has recently been proposed to complement organoid technology, because current PDTO models fall short in replicating tumour micro‐structures and their associated TME.^37^ The emerging concept of ‘tumour assembloids’ emphasizes the interactions among various cells, especially cancer cells and tumour‐associated cells, within a 3D space, aiming to create PDTOs that more authentically mimic the TME. For instance, a bladder assembloid was produced by co‐culturing stem cells and stromal cells. This multilayer approach also develops malignant counterpart tumour assembloids. The pathophysiological features of urothelial carcinoma can be reconstituted, which is beneficial for mechanistic studies.^37^ The assembloid method represents a pivotal advancement in the field, enabling a more faithful representation of in vivo features. The main difference between tumour organoid and tumour assembloids models is the way in which cell culture were made. Organoid technology is a stem cell‐based technology where stem cells differentiate into different cell types within self‐assembled 3D cell spheroids. A unique design of the cell culture medium and protocol are needed. On the other hand, assembloid technology is a tissue engineering‐based technology where different cell types are pre‐prepared and then co‐cultured subsequently or simultaneously within a 3D space.^37^ Assembloid technology provides a more straightforward way to assemble heterogenous and mature cell types, such as immune cells. More importantly, this technology allows spatial control of the ratios between cell types. Altogether, the tumour organoid was named earlier than the tumour assembloids, and studies are distinguishing tumour assembloids and tumour organoid technology. Due to the fact that the goal of these studies is generating primary tumour mimetics to replace animals for cancer therapy, we use PDTO to represent all such tumour mimetics (tumour slices, tumour fragments and 3D cell aggregates containing cancer cells).

### 2.2 PDTOs and tumour assembloids for drug screening

Drug discovery using leukaemia animal models was reported in 1950.^65^ Afterwards, different mouse models for human tumour transplantation, known as PDXs, have been established to predict chemotherapy response.^66^, ^67^ Despite being the gold standard for pre‐clinical drug discovery,^68^ PDXs can solely be evaluated in immunodeficient animals, significantly constraining their relevance in immunotherapy.^69^, ^70^

PDTOs can recapitulate disease‐specific characteristics such as IME,^64^ and encompass more personalized information, including genome, transcriptome, epigenome and proteome.^71^ These properties render PDTOs a more precise model in pre‐clinical drug discovery^72^ and clinical therapy.^73^ For example, Dekkers et al. described a precision method using a functional cystic fibrosis transmembrane conductance regulator (CFTR) assay and primary intestinal organoids.^74^ This method was successfully used to identify and treat patients with rare CFTR mutations who would otherwise have no access to the recently introduced cystic fibrosis drugs.^75^ In addition, PDTOs have been more widely used in evaluating chemotherapeutic and immunologic drugs and clinical trial studies.^76^, ^77^ In 2022, the U.S. President, Joe Biden, signed the legislation that new medicines need not be tested on animals and received approval from the U.S. Food and Drug Administration (FDA). Organizations that promoted this change believe that organoids and other non‐animal protocols, such as artificial intelligence (AI) modelling, should be used more in pre‐clinical drug discovery.^78^

Because of their diversity and editability, PDTOs can be cryopreserved to form a biobank,^79^ customized and scaled up, making them an ideal model for high‐throughput drug screening.^80^ One study showed that a PDTO biobank established from metastatic gastrointestinal cancer patients could summarize the clinical response to regorafenib, palbociclib, taxane, cetuximab and TAS‐102 which can be applied in personalized medicine plans.^81^ A study using a heterogeneous colorectal cancer PDTO biobank and high‐content imaging technology successfully discovered MCLA‐158, a bispecific antibody exhibiting therapeutic properties that specifically trigger epidermal growth factor receptor degradation in cancer stem cells.^82^ Recently, lung cancer organoids were applied to test the sensitivity of multiple chemotherapeutic and targeted drugs. The result showed that the overall accuracy rate of clinical efficacy was as high as 83.3%, the largest sample scale of clinically targeted drugs and chemotherapy drug efficacy tests.^36^

Based on the ability to maintain the TME and the potential to form a biobank, PDTOs are an essential impetus for the upcoming era of individualized precision therapy. Unlike the PDX model or patient‐derived explants (PDE) model,^83^ PDTO has the advantage of being more editable and expandable. PDTO can be personalized and largely designed to meet complex clinical challenges based on race, gender, age, disease and other factors. Therefore, PDTOs have become a promising research model for identifying drug targets and prognostic markers.^84^ With advances in PDTO technology and the establishment of extensive biobanks containing various cancer types, coupled with the utilization of cutting‐edge analysis techniques like high‐content imaging and widefield live‐cell imaging,^85^ PDTOs have now served as precise models for high‐throughput pre‐clinical drug screening, which have in some ways driven changes in drug testing methods before clinical trials.

PDTO technology has also been combined with other technologies, such as liquid biopsy and circulating tumour cell (CTC) collection.^86^, ^87^ CTCs describe cancer cells that have disseminated from the primary tumours to the metastatic sites,^88^ making these cells ideal biomarkers for clinical prognostic scoring. The Veridex CellSearch platform, designed to capture EpCAM‐positive CTCs, gained FDA approval over a decade ago.^89^ Subsequent advancements in this area led to the development of platforms like FICTION (BioView Ltd), which furthered research into characterizing body fluids in cancer patients.^90^ Unfortunately, the CTC counting is not entirely successful in the clinical prognosis due to an extremely low CTC density in the circulation and a significant deviation in CTC capturing. More importantly, the limitation of CTC expansion prevents the further applications of CTC sorting.^91^, ^92^ In 2014, a 3D co‐culture model using a microfluidic device showed the potential of cultivating CTC spheroids.^93^ In another similar endeavour, researchers established various prostate cancer CTC organoids using 3D Matrigel culture, which can be used as genetically manipulatable models for drug response.^94^ Recently, researchers utilized diagnostic leukapheresis to enrich CTCs from metastatic prostate cancer patients, yielding nine CTC organoids.^95^

CTCs can be expanded into tumour spheroids using NSG (NOD scid gamma) immunodeficient mice.^96^ The potential of pancreatic cancer‐derived CTC spheroids was investigated as a pre‐clinical model.^97^ While these encouraging results need to be validated in more patients, they suggested that CTC spheroids can be used to identify patient‐specific disease characteristics, such as phenotype, mutation status and treatment susceptibility. Therefore, CTC‐derived xenograft (CDX) models also promise valuable models.^98^ A study explored the genetic characterization of a unique neuroendocrine transdifferentiation prostate CDX model. The CDX and the derived cell line retained 16% of the primary tumour mutations, 56% of CTC mutations and 83% of primary tumour copy‐number aberrations.^99^ In another study, researchers cultured three CDX models and one CDX‐derived cell line using CTCs from non‐small cell lung cancer patients, outlining patient tumour histology and response to platinum‐based chemotherapy, which mirrored the patient's clinical progression and reflected the patient's response.^100^

Biomaterials and oncology experts have collaboratively explored structured substrates for capturing and growing CTC organoids^101^ and following drug screening.^87^, ^102^ A family of structured patterns, named binary colloidal crystals (BCCs), have unique properties to attract and control tumour cell adhesions.^103^, ^104^, ^105^ Combining optimal BCC and designed CTC medium, researchers have demonstrated that hundreds of CTC organoids could be amplified within 1 month. Similar to the assembloid technology, CTCs and cancer‐associated cells are ‘self‐assembled’ into spheroids on the BCCs. This technology has been applied in pancreatic ductal adenocarcinoma,^87^ head and neck cancers,^102^ small cell lung cancers,^86^ soft tissue sarcoma,^106^ paediatric gliomas^107^ and thymic malignancies.^108^ CTC‐derived organoids are more accessible to prepare than the tumour tissue biopsy‐derived organoids. CTCs are collected from a cancer patient's blood, which is low invasional and time‐efficient.

Researchers have created vascularized tumour assembloids by employing endothelialized microvessels within the tumour spheroids. This complex model approximates the drug penetration pattern observed in hyperthermic intraperitoneal chemotherapy within the tumour nodules while maintaining gene expression patterns akin to those of their parental xenografts.^43^ Nevertheless, owing to the complexities associated with tumour assembloid models, a considerable journey remains before their widespread application in individualized drug detection and screening.

The advancement of technologies in the biomedical field, such as AI^109^ and gene sequencing,^110^ is expected to facilitate the widespread application of PDTOs. Various genetic testing techniques and AI algorithms' assistance have contributed significantly to identifying and classifying PDTOs.^109^, ^111^ Of particular significance is the advancement of AI, which substantially enhances the efficiency and precision of PDTO screening methods.^112^ They will aid in elucidating cancer organoids’ effectiveness and clinical applicability for individual‐level drug response evaluation in clinical precision medicine. The above‐mentioned achievements signify that the establishment of PDTOs is a crucial foundation for personalized and precise medicine, as well as for high‐throughput drug screening and the development of new therapeutics.

## 3 PDTOS IN IMMUNOTHERAPY

The human immune system employs diverse mechanisms, such as humoral immunity and specific immunity, to eliminate tumour cells. However, several of these mechanisms are suppressed in the TME during tumorigenesis.^113^ For instance, hypoxia,^114^ epigenetic modifications^115^ and translational regulation^116^ in the TME also can regulate immune escape and promote tumour development. Researchers have discovered that the dynamic interactions between the neoplastic cells and non‐neoplastic host components within the TME can affect carcinogenesis,^117^ tumour metastasis,^118^ cancer progression^119^ and drug resistance of cancer cells.^120^

The Norwegian Nobel Committee recognized immune checkpoint therapy as an auspicious approach for cancer therapy in 2018 (James Allison and Tasuku Honjo: discovery of cancer therapy by inhibiting negative immune regulation, the 2018 Nobel Prize in Physiology or Medicine). Immunotherapy holds great potential in bolstering the immune system's capacity to identify neoplastic cells and activate an immune response against cancers or augment an existing response directed at the tumour cells.^121^ Therefore, various immunotherapy, including oncolytic viruses,^122^ immune checkpoint inhibitors (ICIs),^123^ cellular immunotherapies such as chimeric antigen receptor‐T cell (CAR‐T) therapy,^124^ T cell receptor‐engineered T cell (TCR‐T) therapy,^125^ bulk tumour‐infiltrating lymphocyte (TIL) therapy,^126^ as well as therapies targeting pattern recognition receptors,^127^ have been employed in current clinical practice.

**FIGURE 3:**
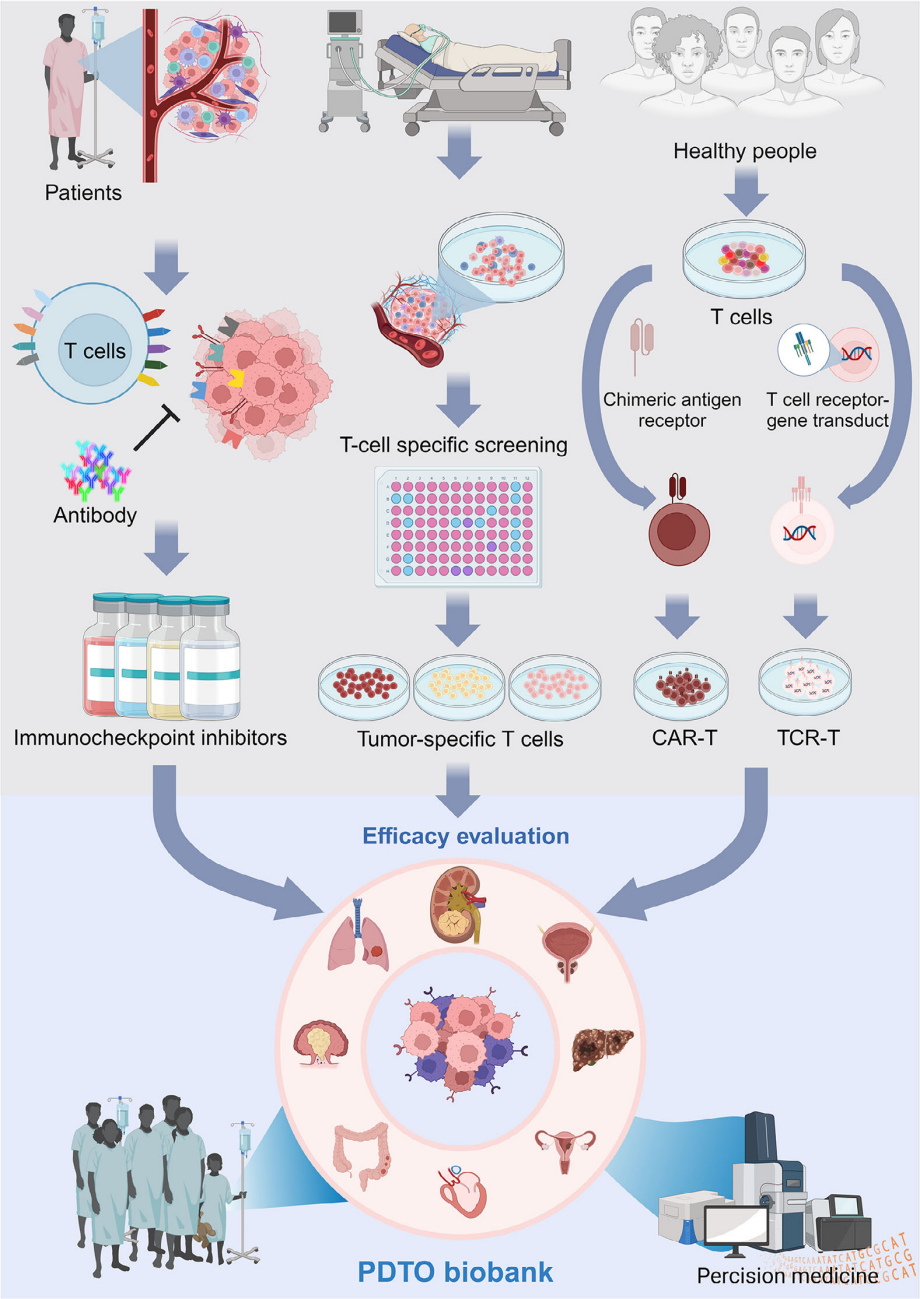
PDTOs can be cancer avatars for predicting immunotherapy regimens, including ICI and immune cell‐based therapies. High‐quality PDTOs should contain multiple cell types, including immune cells. A tissue‐mimicking TME is also critical for immunotherapy evaluation.

While ICIs unblock the immune cell−tumour interactions and awaken the autologous immune system, cell therapies replenish bioengineered immune cells and strengthen the weak immune system of cancer patients. As the immune system's role in cancer immunotherapy is widely recognized, the inadequacy of cell culture and animal models applied in immunotherapy research is becoming increasingly apparent.^2^ Consequently, it is imperative to develop efficient research models incorporating immune cells and other crucial TME components to evaluate the efficacy of immunotherapy interventions (Figure 3). The following sections summarize ICI and immune cell therapies.

### 3.1 Immune checkpoint inhibitors

The immune system constitutes the cornerstone of defence against cancer initiation and progression; however, specific characteristics of neoplastic cells evading immune surveillance contribute to developing the malignant disease.^128^ Currently, ICIs such as those encompassing programmed cell death protein 1 (PD‐1) inhibitors,^129^ cytotoxic T‐lymphocyte antigen 4 inhibitors^130^ and T‐cell immunoglobulin, and mucin domain 3 inhibitors,^131^, ^132^ have gradually established themselves as standard‐of‐care interventions across multiple tumour types. While these ICI treatments have demonstrated considerable efficacy, their effectiveness relies heavily on the individual patient characteristics in the clinical practice.^133^, ^134^

There is still a lack of validated predictive markers for immunotherapy,^135^, ^136^ and suitable models for predicting ICI therapy. Studies have reported that the programmed death‐ligand 1 (PD‐L1) positivity rate, or tumour proportion score, can partially predict the responses to PD‐1 or PD‐L1 drugs,^137^ but there is no consensus among clinicians regarding this approach. Other factors, such as specific immune transcriptional signatures,^138^ spatial distribution of immune cells^139^ and spatial expression of immune elements^140^, ^141^ within the tumour milieu, have been acknowledged to dictate sensitivity/resistance to ICI rather than the PD‐L1 expression. However, they also lack effective, validated models to achieve common acceptance in clinical research. Consequently, it becomes paramount to develop appropriate in vitro models, such as PDTO models, for understanding disease pathology and predicting the efficacy of drug treatments.

PDTOs offer key TME components, including T cells, natural killer (NK) cells and tumour stromal cells, offering an invaluable platform for investigating the effects of ICIs. However, a significant hurdle arises from the difficulty of promptly procuring clinical biopsy tissues and generating PDTOs. Despite these limitations, PDTOs fully represent TME and exhibit superior drug prediction accuracy for ICIs compared to traditional cell culture models.^36^ Even though the current pre‐clinical trial application of PDTO is limited and lacks immediate patient impact, along with the accumulation of research and the systematic establishment of the PDTO biobank, it can be applied to large‐scale prediction trials of immunotherapies, potentially offering a lifeline to critically ill patients.^142^ Furthermore, establishing PDTO biobanks allows researchers and clinicians to study the genetic mutations and TEM traits of cancers systematically. With the large sample size of PDTOs serving as a drug screening platform, researchers have more opportunities to identify new genetic markers that affect the efficacy of immunotherapeutic drugs and explore possible cellular immune mechanisms.^143^ Such endeavours also advance the use of PDTOs in pre‐clinical trials of immunotherapies from a fundamental research perspective. This data‐driven approach can overcome individual variations in immunotherapy and is of great value in guiding clinical use. Overall, PDTOs with IME provide a platform for pre‐clinical drug screening and the mechanistic exploration of various cancers using ICIs.

### 3.2 Adoptive cell therapy

(ACT) is a remarkably personalized form of cancer immunotherapy in which lymphocytes are infused back into the body to orchestrate anti‐tumour, anti‐viral or anti‐inflammatory responses.^144^ ACT can be performed using either host cells naturally endowed antitumor reactivity, such as bulk TILs, or genetically engineered host cells expressing anti‐tumour T cell receptors (TCRs) or chimeric antigen receptors (CARs).^145^ Several promising cellular immunotherapies and prospects for PDTO applications are summarized below.

#### 3.2.1 TIL therapy

Bulk TILs represent a subset of infiltrating lymphocytes isolated from tumour tissues.^146^ Some lymphocytes are T cells that can detect tumour‐specific mutant antigens, marking them as formidable immune cells capable of infiltrating tumours; they signify the body's specific immune response against tumour cells, yielding remarkable lethality.^147^, ^148^ Effective cells of TIL therapy are naturally selected and enriched populations with a high proportion and rich diversity of tumour‐specific T cells, which have the advantages of multi‐targeting, high tumour tropism, infiltration capacity and low side effects.^149^

The meticulous execution of the in vitro T‐cell screening procedures is an indispensable factor influencing therapeutic outcomes.^150^ Currently, the widely accepted method entails the isolation and amplification of TILs from a patient's tumour mass, followed by co‐culturing with the patient's tumour cells and amplified T lymphocytes to selectively identify TILs with specific tumour recognition abilities.^149^ However, the challenge persists in using tissue samples from the patients solely for homologous TIL screening.^151^ Obtaining fresh tumour samples poses a difficulty, and the associated cost and time constraints during cell expansion further compound the challenges. In addition, factors like the unpredictable in vivo retention time and TME suppression on the reintroduced T cells limit their clinical utility.^152^, ^153^

Researchers have explored the feasibility of heterologous tumour cell screening in TIL therapy due to the urgency in clinical timelines. While homologous screening yields TILs with high tumour‐specific recognition abilities, it often restricts their broad‐spectrum applicability; conversely, heterogeneous screening can make TIL obtain broad‐spectrum efficacy but may lead to immune rejection across individuals. Herein lies the potential of a comprehensive PDTO biobank in addressing the limitations of TIL therapy. To this end, researchers are pursuing the generation of TILs possessing personalized recognition and cytotoxic capabilities through in vitro methods.^154^, ^155^ Specifically, one proposed strategy involves co‐culturing peripheral blood lymphocytes and tumour organoids to generate tumour‐reactive T cells.^156^ Furthermore, the concept of ‘super T cells’ with exceptional abilities to sustain high levels of specific recognition and cytotoxicity towards tumour organoids is under investigation. However, these aspects require thorough investigation, especially in a large pool of PDTO samples. It should be emphasized that for TIL screening for specific tumour killing, especially for heterologous screening, the PDTO biobank is an ideal platform to help identify the most promising in vivo immunotherapy candidates. The improved TME and the diversity of PDTOs add to the safety of the treatment for different individuals and also provide a better research environment for exploring the therapy mechanism.

#### 3.2.2 TCR‐T therapy

TCR‐T therapies are grounded in their ability to recognize tumour‐specific antigens located on the cell membrane surface or originating intracellularly.^157^ These specialized T cells can identify antigens within tumours and are more likely to breach solid tumours’ robust defences.^158^, ^159^ A notable advantage of TCR‐T therapies is that the TCR‐T cells are naturally expressed in the human body and fully humanized, thereby mitigating the risk of immune rejection. In addition, TCR‐T cells possess immune memory and can persist in vivo for an extended period, amplifying their therapeutic efficacy.

The principle of TCR‐T therapy is to increase the affinity of TCRs towards tumour‐associated antigens and the fighting power of immune cells by transducing CARs (fusion antigen binding domain and T cell signalling structural domain) or TCRα/β heterodimers into conventional T cells. These modifications enable T lymphocytes to efficiently re‐recognize target cells and exert strong anti‐tumour immune effects in vivo.^150^ However, there are particular challenges associated with TCR engineering modifications. First, endogenous TCRs remain in T cells, suppressing surface expression and generating mismatched prosthetic TCRs, leading to decreased transgenic TCR function and potentially to self‐reactivity or graft‐versus‐host disease.^160^ Second, viral transduction leads to random integration of transgenes, and non‐viral transduction presents safety issues with at least semi‐random gene integration.^161^, ^162^ All these issues limit its clinical efficiency.

TCR‐T therapies offer a broader range of antigen recognition, which makes it adaptable to a larger patient population in broad‐spectrum treatments.^163^ However, the clinical efficacy of TCR‐T therapies in individualized therapy remains limited.^159^ The intricate in vivo biological environment challenges accurately replicating the myriad possibilities in vitro. Leveraging the advantages of PDTOs and an evolving culture system, the PDTO library emerges as a crucial resource for TCR‐T therapy.^164^ Specifically, for the clinical challenge of TCR‐T therapies, the editability of PDTO means that it is possible to construct tumour organoids of different types and with different tumour antigenic profiles. Meanwhile, the study of the antigen recognition profiles of TCR‐T is inherently more limited.^157^ The extensive PDTO‐based biobank offers an unmatched platform for pre‐clinical testing of unexplored TCR‐T therapies, which undoubtedly gives physicians and patients more confidence for clinical practice. Researchers have successfully propagated PDTOs with native embedded immune cells (i.e. T, B, NK and macrophages) to reconstruct TME effectively. Notably, PDTO‐derived TILs accurately retain the original TCR spectrum.^64^ This provides crucial insights for conducting tumour immunology studies within the TME, thereby expediting research in personalized immunotherapy testing. Undoubtedly, this has shifted researchers’ attention towards the essential need to establish PDTO biobanks, as PDTOs can serve as invaluable pre‐clinical experimental models for TCR‐T screening. By utilizing PDTOs, more crucial data can be obtained to ensure the efficacy and safety of treatments for individual patients before proceeding to formal clinical trials. Overall, pre‐clinical drug screening models based on PDTOs, including PDTO‐based animal transplantation models and 3D drug‐detection models, promise comprehensive insights for the pre‐clinical assessment of TCR‐T therapies.

#### 3.2.3 CAR‐T therapy

CAR‐T therapy is based on genetically modifying CARs to target tumour cell antigen recognition and kill tumour cells.^165^ The CARs, integral components of CAR‐T cells, are protein receptors empowering T cells to exclusively recognize specific proteins (antigens) on the surface of tumour cells; T cells expressing CAR identify and bind these tumour antigens, instigating an attack on tumour cells.^166^

CAR‐T cells are primarily derived from autologous T cells, which are extracted and genetically engineered before undergoing extensive in vitro amplification, ultimately yielding billions of T cells. These cells are then reinfused into the patient for immunotherapeutic purposes.^167^ This direct action bypasses the antigen presentation, rendering CAR‐T therapy more suitable for haematologic tumours with prominent surface antigen exposure.^168^ In solid tumour treatment, CAR‐T therapies are stuck in a rut now. The main problems of CAR‐T therapies are their severe side effects and off‐target effects.^168^, ^169^ Moreover, the transfusion of immune cells back into the body can result in a severe immune storm, leading to unpredictable therapeutic outcomes.^170^ Considering these concerns, researchers have proposed the need for PDTO models for immunotherapies.^143^ Jacob et al. generated glioblastoma organoids (GBOs) from surgically resected patient tumour tissue and outlined a protocol for investigating patient‐specific responses to immunotherapy through co‐culturing GBOs with CAR‐T cells.^142^

Given the current feasibility of establishing a large PDTO biobank, it offers the possibility of simulating the in vitro effects of numerous immunotherapies. However, the problem is that under autologous therapies, the autologously constructed PDTO model still cannot promptly respond to the actual situation in vivo; thus, researchers have proposed CAR‐T‐based allogeneic therapies.^171^ Autologous therapy involves in vitro modification and expansion of T cells extracted from the patient, which is often challenging due to the damaged state of the patient's lymphocytes, leading to insufficient numbers of high‐quality T cells and increased time and costs.^172^, ^173^ In contrast, allogeneic therapy retrieves high‐quality T cells from healthy donors, but this requires immune modification to reduce the risk of host anti‐graft rejection. While some clinical trials have positive outcomes,^174^, ^175^ concerns about safety persist for the widespread application of universal CAR‐T therapies since they are allogeneic‐derived T cells.

The PDTO biobank not only meets the TME in various pathological conditions, but can also modify a wide variety of tumour‐specific antigens to verify the targeting and safety of CAR‐T therapies. Furthermore, integrating allogeneic therapy with the foundation of a PDTO biobank has the potential for in vitro screening of healthy and high‐quality T cells. Modifying these T cells aims to enhance their tumour‐specific targeting ability while reducing the risk of immune rejection. Therefore, with the extensive construction of PDTOs in the early stage, although it cannot help patients to evaluate the efficacy of CAR‐T (such as universal CAR‐T) promptly in the present time, it undoubtedly provides necessary resources and value for in vitro screening of universal CAR‐T cells.

#### 3.2.4 Other immune cells‐based therapies

Other immune cells‐based therapies have been explored due to their distinct advantages, including NK cells,^176^ gamma delta T cells (γδT),^177^ CD3 activated killer cells,^178^ cytokine‐induced killer cells (CIK),^179^ dendritic cell‐cytokine induced killer cells (DC‐CIK)^180^ and lymphokine‐activated killer cells.^181^, ^182^ These cell‐based therapies have shown continuous progress in efficacy, specificity and reduction of side effects. However, considering the challenges posed by clinical trials compared to mainstream T‐cell therapies, other immune cell therapies typically require more time and extensive testing models to assess their applicability.

Among these immune cells, NK cells are the frontline defence in the human immune system, capable of directly detecting and destroying exogenous threats and tumour cells without prior activation.^183^ Notably, the success of CAR‐T therapy has driven the evolution of CAR‐NK cell therapy, emerging as a highly effective anticancer immunotherapy strategy.^184^ The FDA in the United States has already approved several NK cell therapies, such as FT536 (Fate Therapeutics 536), which is derived from allogeneic, multiply‐engineered iPSCs.^185^ In 2021, China has also approved a clinical trial application for a CAR‐NK injection targeting mesothelin for treating advanced epithelial ovarian cancers.^186^

**FIGURE 4:**
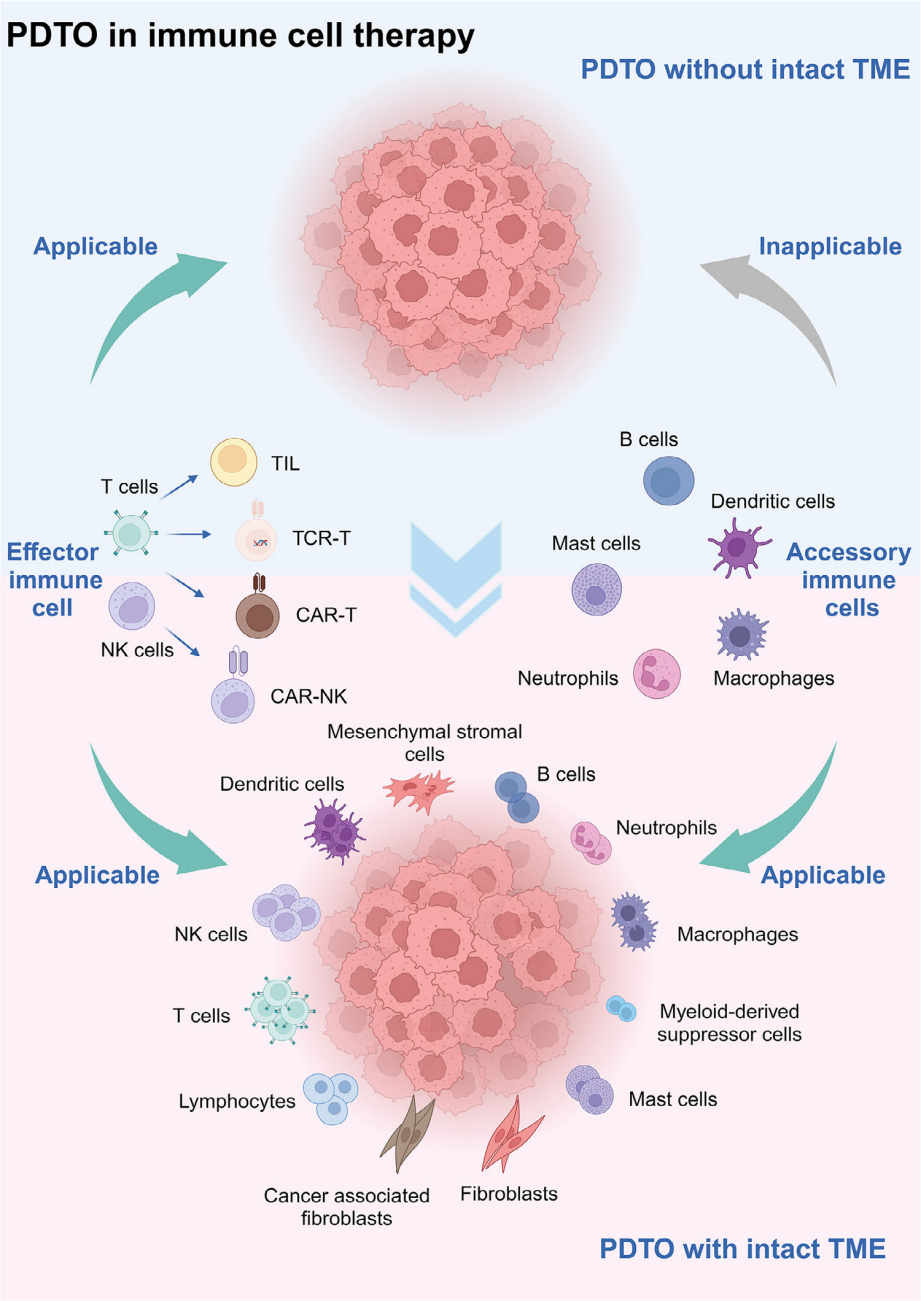
Future PDTOs in immune cell therapies. PDTOs without intact TME are unsuitable for pre‐clinical efficacy testing of accessory immune cell therapies, mainly because they lack effector immune cells such as T and NK cells. However, as technology advances, future PDTOs with intact TME will likely serve as a reliable efficacy screening platform for all immune cell therapies.

Cell‐based strategies focus on improving the weakened immune system against cancers by employing advanced biotechnology to collect, modify and expand a patient's immune cells. However, the clinical application of cell therapy requires extensive trials due to individual differences and the distinct roles of various immune cells in the immune system. The current PDTO technology fails to fully replicate the original TME, limiting its application in accessory immune cell therapies. Most current PDTOs lack sufficient immune cell components, such as lymphocytes that can be continuously regenerated and transformed, which could result in an unreliable prediction using accessory immune cell therapies such as dendritic cells. A future PDTO with immune cells is illustrated in Figure 4.

## 4 PDTOS IN CLINICAL APPLICATION

3D cell culture technology is superior to classical 2D cell culture in predicting drug response.^187^ PDTOs are patient‐derived aggregates that can be grown in 3D and maintain self‐renewal pluripotency and lineage‐specific differentiation.^17^ Therefore, they are thought to maintain patients’ heterogeneity and characterization compared to conventional cell lines, and they have been used for drug response screening.^76^ The European Union Drug Regulating Authorities Clinical Trials Database (EudraCT) and ClinicalTrials.gov, two comprehensive clinical trial search platforms, have been accessed for some clinical trials involving PDTOs. It demonstrates that researchers are now widely aware of the value of PDTOs in reflecting individual characterization, drug tests and mechanistic studies.

As of 16 January 2024, five clinical trials involving the application of PDTO were registered in EudraCT (Table 1). First initiated in 2016, the clinical trials mainly involved drug tests of PDTO in chemical origins, such as palbociclib, oxaliplatin, and biological/biotechnological origins, such as pembrolizumab and trastuzumab. More clinical trial information was displayed on ClinicalTrials.gov (Table 2), with 58 registered clinical trials involving PDTO. The main research scope consists of the establishment, characterization, evaluation, sequencing and drug test of PDTOs, of which only three have been completed; it includes (1) Duke University completed an establishment of rectum cancer PDTO (NCT04371198), (2) the Mayo Clinic completed a drug response testing in PDTOs derived from biliary tract cancer (NCT04072445) and (3) the Chinese University of Hong Kong completed sequencing of meningioma PDTOs (NCT04478877).

**TABLE 1:** Clinical trials involving the application of PDTO in EudraCT.

EudraCT number	Full title of the trial	Diseases	Application	Start date
2014‐003811‐13	Selecting cancer patients for treatment using Tumor Organoids, the SENSOR study	Colorectal cancer and non‐small cell lung cancer	Drug test	2016‐06‐16
2020‐003697‐52	Systemic Neoadjuvant and adjuvant Control by Precision medicine in rectal cancer (SYNCOPE)—approach on high‐risk group to reduce metastases	Rectal cancer	Drug test	2020‐09‐16
2020‐003395‐41	An open‐label single‐arm interventional phase 2 study to investigate the outcome of individualized treatment based on pharmacogenomic profiling and ex vivo drug sensitivity testing of patient‐derived organoids in patients with metastatic colorectal cancer	Metastatic colorectal cancer	Drug test	2021‐01‐15
2021‐001181‐38	Anti‐PD‐1, Capecitabine, and Oxaliplatin for the first‐line treatment of dMMR esophagogastric cancer (AuspiCiOus‐dMMR): a proof‐of‐principle study	Gastroesophageal cancer	Drug test	2021‐07‐29
2021‐006276‐16	PaTcH Trial: A phase 2 study to explore primary and emerging resistance mechanisms in patients with metastatic refractory pancreatic cancer treated with trametinib and hydroxychloroquine.	Advanced pancreatic cancer	Drug test	2022‐03‐09

Abbreviation: EudraCT, European Union Drug Regulating Authorities Clinical Trials Database. https://eudract.ema.europa.eu/.

**TABLE 2:** Clinical trials involving the application of PDTO in ClinicalTrials.gov.

ClinicalTrials.gov number	Study title	Diseases	Application	First posted
NCT02732860	Personalized Patient Derived Xenograft (pPDX) Modeling to Test Drug Response in Matching Host (REFLECT)	Breast cancer, colorectal cancer, high‐grade serous ovarian cancer and other select tumour types	Drug test	2016‐04‐11
NCT03283527	Organoid Based Response Prediction in Esophageal Cancer (RARESTEM/Org)	Esophageal cancer	Drug test	2017‐09‐14
NCT03453307	Drug Sensitivity Correlation Between Patient‐Derived Organoid Model and Clinical Response in NSCLC Patients	Non‐small cell lung cancer	Drug test	2018‐03‐05
NCT03500068	Establishing Organoids From Metastatic Pancreatic Cancer Patients, the OPT‐I Study. (OPT‐1)	Metastatic pancreatic cancer	Establishment and analysis	2018‐04‐17
NCT03544047	Clinical Study on Drug Sensitivity Verification or Prediction of Therapy for Breast Cancer by Patient‐Derived Organoid Model	Breast cancer	Drug test	2018‐06‐01
NCT03655015	Patient‐derived Organoid Model and Circulating Tumor Cells for Treatment Response of Lung Cancer	Lung neoplasm	Drug test	2018‐08‐31
NCT03764553	Liposomal iRInotecan, Carboplatin or oXaliplatin for Esophagogastric Cancer (LyRICX)	Esophagogastric cancer	Drug test	2018‐12‐05
NCT03979170	Patient‐derived Organoids of Lung Cancer to Test Drug Response	Lung cancer	Drug test	2019‐06‐07
NCT03990675	Evaluation and Comparison of the Growth Rate of Pancreatic Cancer Patient‐derived Organoids	Pancreatic cancer	Protocol evaluation	2019‐06‐19
NCT04072445	Trifluridine/Tipiracil and Irinotecan for the Treatment of Advanced Refractory Biliary Tract Cancer	Bile duct carcinoma, gallbladder carcinoma	Drug test	2019‐08‐28

ClinicalTrials.gov number	Study title	Diseases	Application	First posted
NCT04219137	Molecular Characteristics of Gastroesophageal Adenocarcinoma (MOCHA): A Prospective Feasibility Study (MOCHA)	Esophagogastric adenocarcinoma	Drug test	2020‐01‐06
NCT04279509	Selecting Chemotherapy With High‐throughput Drug Screen Assay Using Patient Derived Organoids in Patients With Refractory Solid Tumours (SCORE)	Head and neck squamous cell carcinoma, colorectal cancer, breast cancer, epithelial ovarian cancer	Drug test	2020‐02‐21
NCT04371198	Patient‐Derived Organoids for Rectal Cancer	Rectum cancer	Establishment	2020‐05‐01
NCT04478877	Establishment and Characterization of Meningioma Patient‐derived Organoids	Meningioma	Sequencing	2020‐07‐21
NCT04555473	Translational Analysis In Longitudinal Series of Ovarian Cancer ORganoids (TAILOR)	Epithelial ovarian cancer	Sequencing and Drug test	2020‐09‐18
NCT04611035	Q‐GAIN (Using Qpop to Predict Treatment for GAstroIntestinal caNcer)	Gastrointestinal cancer	Drug test	2020‐11‐02
NCT04655573	A Pilot Study of a Micro‐Organosphere Drug Screen Platform to Lead Care in Advanced Breast Cancer	Advanced breast cancer	Drug test	2020‐12‐07
NCT04768270	The Culture of Ovarian Cancer Organoids and Drug Screening	Ovarian cancer	Sequencing and Drug test	2021‐02‐24
NCT04865315	A Living Tissue Bank of Patient‐Derived Organoids From Glioma Tumors (HiLoGlio)	Glioma	Sequencing	2021‐04‐29
NCT04868396	Patient‐derived Glioma Stem Cell Organoids	Glioblastoma	Drug test	2021‐04‐30

ClinicalTrials.gov number	Study title	Diseases	Application	First posted
NCT04906733	Cetuximab Sensitivity Correlation Between Patient‐Derived Organoids and Clinical Response in Colon Cancer Patients	Colon cancer	Drug test	2021‐05‐28
NCT04927611	Single‐cell Sequencing and Establishment of Models in Neuroendocrine Neoplasm	Neuroendocrine neoplasm	Establishment	2021‐06‐16
NCT05007379	Cohort Study to Determine the Antitumor Activity of New CAR‐macrophages in Breast Cancer Patients' Derived Organoids (CARMA)	Breast cancer	Drug test	2021‐08‐16
NCT05024734	Guiding Instillation in Non Muscle‐invasive Bladder Cancer Based on Drug Screens in Patient Derived Organoids	Bladder cancer	Drug test	2021‐08‐27
NCT05038358	Tumor Immune Microenvironment Involvement in Colorectal Cancer Chemoresistance Mechanisms (CRC‐ORGA 2)	Colorectal cancer	Establishment	2021‐09‐09
NCT05092009	Lung Cancer Organoids and Patient Derived Tumor Xenografts	Lung cancer	Drug test	2021‐10‐25
NCT05136014	Evaluation of the Response to Tyrosine Kinase Inhibitors in Localized Non‐small Cell Lung Cancer (NSCLC) Patients With EGFR Mutation in a Patient‐derived Organoid Model (OS‐TUMOVASC)	Non‐small cell lung cancer	Drug test	2021‐11‐26
NCT05175326	Study on the Consistency Evaluation of Organoids Used in the Clinical Treatment of Ovarian Cancer With Anti‐tumor Drugs	Ovarian cancer	Drug test	2022‐01‐03
NCT05177432	Quadratic Phenotypic Optimisation Platform (QPOP) Utilisation to Enhance Selection of Patient Therapy Through Patient‐Derived Organoids in Breast Cancer	Breast cancer	Drug test	2022‐01‐04

ClinicalTrials.gov number	Study title	Diseases	Application	First posted
NCT05183425	Patient‐derived Organoids Predicts the Clinical Efficiency of Colorectal Liver Metastasis	Primary colorectal cancer and liver metastases	Drug test	2022‐01‐10
NCT05196334	Pharmacotyping of Pancreatic Patient‐derived Organoids	Pancreatic cancer	Sequencing and Drug test	2022‐01‐19
NCT05203549	Consistency Between Treatment Responses in PDO Models and Clinical Outcomes in Gastric Cancer	Gastric cancer	Drug test	2022‐01‐24
NCT05290961	The Culture of Advanced or Recurrent Ovarian Cancer Organoids and Drug Screening	Ovarian neoplasms	Drug test	2022‐03‐22
NCT05304741	The Culture of Advanced/Recurrent/Metastatic Colorectal Cancer Organoids and Drug Screening	Colorectal cancer	Drug test	2022‐03‐31
NCT05317221	Developing Breast (Cancer) Organoids	Breast cancer	Establishment	2022‐04‐07
NCT05351983	Patient‐derived Organoids Drug Screen in Pancreatic Cancer	Pancreatic cancer	Drug test	2022‐04‐28
NCT05351398	The Clinical Efficacy of Drug Sensitive Neoadjuvant Chemotherapy Based on Organoid Versus Traditional Neoadjuvant Chemotherapy in Advanced Gastric Cancer	Advanced gastric carcinoma	Drug test	2022‐04‐28
NCT05378048	Patient‐derived‐organoid (PDO) Guided Versus Conventional Therapy for Advanced Inoperable Abdominal Tumors	Inoperable or metastatic abdominal tumours	Drug test	2022‐05‐17
NCT05384184	Next Generation Pre‐clinical Model for Colorectal Cancer Metastases and Hepatocellular Carcinomas (BORG)	Colorectal cancer metastases and hepatocellular carcinomas	Establishment and evaluation	2022‐05‐20
NCT05401318	Tailoring Treatment in Colorectal Cancer (TargetCRC)	Colorectal cancer	Drug test	2022‐06‐02
ClinicalTrials.gov number	Study title	Diseases	Application	First posted
NCT05464082	Functional Precision Oncology to Predict, Prevent, and Treat Early Metastatic Recurrence of TNBC (TOWARDS‐II)	Breast cancer	Drug test	2022‐07‐19
NCT05634694	Study on Consistency Evaluation for Drug Sensitivity of Patient‐Derived Organoid Model From Cholangiocarcinoma Patients	Cholangiocarcinoma	Drug test	2022‐12‐02
NCT05644743	Clinical Transformation of Organoid Model to Predict the Efficacy of GC in the Treatment of Intrahepatic Cholangiocarcinoma	Intrahepatic cholangiocarcinoma	Sequencing and Drug test	2022‐12‐09
NCT05652348	Response Prediction of Hyperthermic Intraperitoneal Chemotherapy in Gastro‐Intestinal Cancer	Gastric cancer, colon cancer, peritoneal carcinomatosis	Sequencing and Drug test	2022‐12‐15
NCT05669586	Organoids Predict Therapeutic Response in Patients With Multi‐line Drug‐resistant Non‐small Cell Lung Cancer	Non‐small cell lung cancer	Drug test	2023‐01‐03
NCT05725200	Study to Investigate Outcome of Individualized Treatment in Patients With Metastatic Colorectal Cancer (EVIDENT)	Metastatic colorectal cancer	Drug test	2023‐02‐13
NCT05734963	ORganoid GeneratioN Study for Cancer (ORIGINS)	Cancer	Sequencing	2023‐02‐21
NCT05772741	Grafts of GSCs Into Brain Organoids for Testing Anti‐invasion Drugs	Glioblastoma	Drug test	2023‐03‐16
NCT05832398	Precision Chemotherapy Based on Organoid Drug Sensitivity for Colorectal Cancer	Colorectal cancer	Drug test	2023‐04‐27
NCT05883683	Molecular Study and Precision Medicine for Colorectal Cancer	Advanced or recurrent colorectal cancer	Sequencing and Drug test	2023‐06‐01
NCT05913141	PDO/PDO‐TIL/PDOTS for Drug Screen	Liver cancer	Drug test	2023‐06‐22

ClinicalTrials.gov number	Study title	Diseases	Application	First posted
NCT05927298	Province of Ontario Strategy for Personalized Management of Pancreatic Cancer Trial (ProsperPanc)	Pancreas cancer	Drug test	2023‐07‐03
NCT05955196	Test of CD47‐SIRPα Inhibitors on the Immune Microenvironment Colon Cancer (MACROSWITCH)	Colon cancer	Drug test	2023‐07‐21
NCT06077591	Prospective Clinical Validation of Next Generation Sequencing (NGS) and Patient‐Derived Tumor Organoids (PDO) Guided Therapy in Patients With Advanced/Inoperable Solid Tumors	Hepatocellular carcinoma colorectal cancer	Drug test	2023‐10‐11
NCT06085404	Patients Derived Organoids in Ovarian Cancer (PICTURE)	Ovarian cancer	Drug test	2023‐10‐17
NCT06102824	Organoid‐based Functional Precision Therapy for Advanced Breast Cancer (ORIENTA)	HER2‐negative advanced breast cancer	Drug test	2023‐10‐26
NCT06155305	Organoids Based Drug Sensitivity in Neoadjuvant Chemotherapy of Breast Cancer (ONAC)	Breast cancer	Drug test	2023‐12‐04
NCT06195150	Overtaking Intra and Inter Tumoral Heterogeneity In Von Hippel‐Lindau Related Renal Cancer (ITHORinVHL)	Von Hippel‐Lindau‐related renal cancer	Drug test	2024‐01‐08

ClinicalTrials.gov: https://classic.clinicaltrials.gov/ct2/home.

Clinical trials using PDTO in immunotherapy are beginning to emerge. For example, a trial was planned to establish PDTO with breast cancer and test CAR‐macrophages’ anti‐tumour activity (NCT05007379). In the foreseeable future, clinical trials and applications of PDTO in immunotherapy will develop rapidly. Overall, the previous clinical trials on PDTO mainly focused on the protocol and evaluation of drug response, while more and more researchers are now focusing on their molecular characteristics and beginning to combine them with basic experiments and clinical uses, such as immunotherapy and molecular mechanism studies.

## 5 FUTURE PDTOS

**FIGURE 5:**
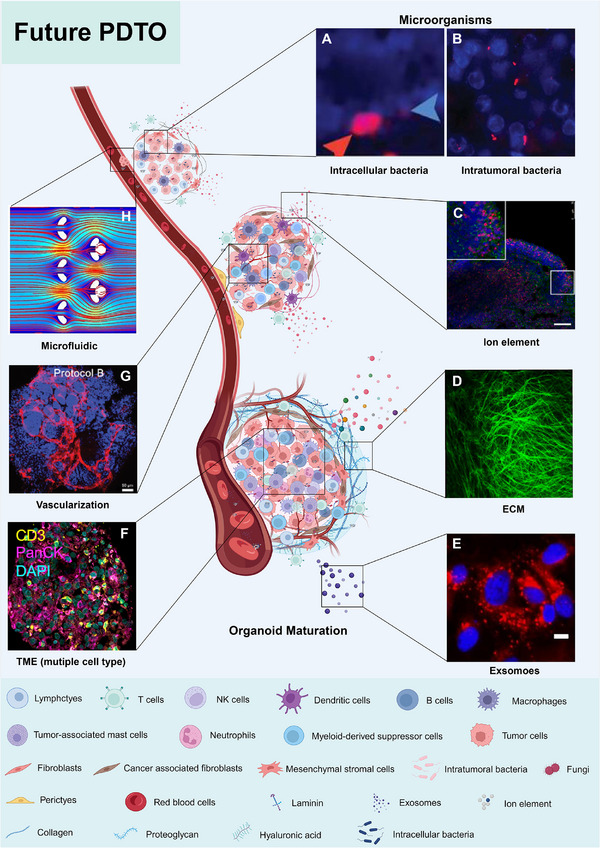
A summary of future and high‐quality PDTOs. Critical components in PDTOs include (A, B) microorganisms (Reproduced with permission from Refs. ^199^ and 204. Copyright 2020 AAAS and Copyright 2017 AAAS), (C) ion elements (Reproduced with permission from Ref. ^205^. Copyright 2021 Elsevier B.V.), (D) ECM (Reproduced with permission from Ref. ^206^. Copyright 2022 Springer Nature), (E) exosomes or extracellular vesicles (Reproduced with permission from Ref. ^207^. Copyright 2020 Ivyspring International Publisher), (F) TME (Reproduced with permission from Ref. ^64^. Copyright 2018 Elsevier Inc.), (G) angiogenesis (Reproduced with permission from Ref. ^208^. Copyright 2022 John Wiley & Sons, Inc.) and (H) fluid environment and mechanical stimulation (Reproduced with permission from Ref. ^60^. Copyright 2014 AIP).

With the rise of immunotherapy, researchers need a model close to the primary TME for clinical prediction.^47^ Patient‐relevant pre‐clinical models, including PDTO, PDX, PDE and tumour spheroids, were widely reported.^188^, ^189^ To varying degrees, they compensate for differences with the in vivo phenotype, microenvironment and genomics.^190^ The PDX model remains an essential standard for pre‐clinical testing. In contrast, tumour spheroids, PDE, PDTOs and tumour assembloids show more kindness in reducing the attrition of experimental animals. Undoubtedly, researchers also believe they offer potential as in vitro prediction models.^31^, ^191^ PDTOs retain more of the original tissue's stromal environment and cellular components and can consistently maintain the original IME.^192^ Based on the possibility of open access to fresh tissue samples, the PDE model has greatly facilitated its research in immunological detection^193^ and drug response detection.^194^ However, technical problems exist, such as tissue loss and heterogeneity.^195^ We cannot still construct PDTOs with sufficient tumour‐associated cells, endothelial cells (i.e. blood vessels)^196^ and immune cells.^197^, ^198^ Some tissues, such as the skin and gut, key components, including the microbiome,^199^ ion element^200^ and exosomes,^201^ are also insufficient, significantly impeding the practical application of tumour organoids in basic and clinical research.^202^, ^203^ Despite these limitations, these challenges provide insight into future directions for refining tumour organoid technology (Figure 5).

PDTO has more editability and construction flexibility and can be constructed in multiple ways to meet basic and clinical research needs. The adoption of PDTOs and new analysing technologies is poised to significantly advance drug discovery, which is undoubtedly irreplaceable for the rapid advancement of precision medicine. Indeed, a necessary impetus is the urgent need to complete the construction of a standardized PDTO biobank. Establishing conventional cell culture platforms has taken decades of effort but is no longer sufficient to meet the needs of the rapidly evolving research. At the same time, the PDTO biobank will be an alternative in vitro model for the next generation of basic research.

Another driving force for PDTO research is the need for clinical precision medicine. Mainly, new progress has been made in adoptive T‐cell transfer therapies, including TIL therapy, CAR‐T therapy and TCR‐T therapy. The technological development of PDTOs has not yet reached a point where they can fully recreate an in vitro TME that accurately represents the complete immune cell populations, including T cells, NK cells, DCs, B cells and other tumour‐associated cells. Consequently, the current PDTOs in pre‐clinical testing of cellular immunotherapies remain limited. However, on the other hand, when it comes to studying T cells that directly target tumour cells, PDTOs serve as a valuable model for assessing the efficacy of these immune ‘soldiers’ in attacking tumours.

In 2022, Hu et al. developed the world's first allogeneic CAR‐T cell to treat refractory CD7‐positive haematological malignancies.^174^ In the same year, the clinical application prospect of allogeneic CAR‐T therapy was also reported.^209^ In TCR‐T therapy, researchers successfully transformed immune cells using CRISPR/Cas9 technology. They first looked for specific mutations of cancer cells in the tumour samples and evaluated mutations that are more likely to stimulate T cell immune response. After several rounds of validation, TCRs that can accurately identify cancer cells were found, and their gene sequences were inserted into T cells through CRISPR gene editing. Finally, TCR‐T cells that can specifically recognize cancer cells of almost all patients were constructed. This is the first intersection of the two hot fields of personalized gene editing and cancer immunotherapy.^210^ These studies evaluate the safety and feasibility of phase I clinical trials. There is still a lack of reliable research models to help carry out the pre‐clinical efficacy evaluation of new immunotherapy. The PDTOs hold as a vital solution. Through the establishment of the PDTO biobank, the curative effect can be evaluated before clinical treatment. Combined with gene editing technology^17^, ^211^ and next‐generation sequencing technology,^212^ PDTOs play essential roles in drug discovery and efficacy evaluation, significantly improving new therapies’ clinical safety. The feasibility of PDTO construction formed a solid base for treating the growing clinical needs.

Despite the numerous applications of PDTO technology in drug discovery and clinical drug screening, significant challenges remain. For example, no broad agreed‐upon conceptual and technical framework exists for understanding PDTOs.^213^ Due to variations in research methodologies, the PDTOs developed by various teams may not comprehensively recapitulate the full spectrum of cell types in primary tumours, including non‐parenchymal cells (immune and stromal cells). It may also fail to accurately model the tissue's dynamic phenotypic changes in the chronological state.^214^ Furthermore, the capacity of PDTOs to elucidate the impacts of environmental exposure, biological ageing and vascularization on human organs is constrained. Specifically, vascularizing PDTOs is crucial for research on immune cell infiltration and drug delivery. Recently, this immune‐infiltrated, vascularized kidney organoid‐chip model exhibited surprising predictive results in T‐cell cancer immunotherapy.^58^, ^215^ This is undoubtedly a shot in the arm for organoid research. Another significant challenge is the lack of consensus among research teams regarding optimal methods for constructing PDTOs,^203^ and so does the culture system of PDTOs.^216^ Reproducing PDTOs under varying culture systems in different laboratories has become a major concern among researchers.

Researchers have emphasized the significance of co‐culturing multiple cell types to establish PDTOs and have achieved notable advancements. The introduction of the assembloid concept has marked a significant stride in the evolution of organoid technology.^38^ One aspect that demands further consideration is the absence of microorganisms,^217^, ^218^ exosomes^219^ and ion elements^220^ in PDTOs, which are vital components of the TME. Bacteria have been detected in human tumours for over a century but are often ignored. In recent years, researchers have realized the significant impact of microorganisms on tumour initiation and progression.^221^, ^222^ It has been confirmed that microorganisms, including tumours, are distributed all over the human body. Most of these bacteria in tumours are ‘intracellular bacteria’.^204^ In a study on breast cancer, many unique ‘intracellular bacteria’ affected tumour metastasis and colonization.^223^ This finding inspires researchers to realize that PDTOs need to consider the influence of microorganisms.

**FIGURE 6:**
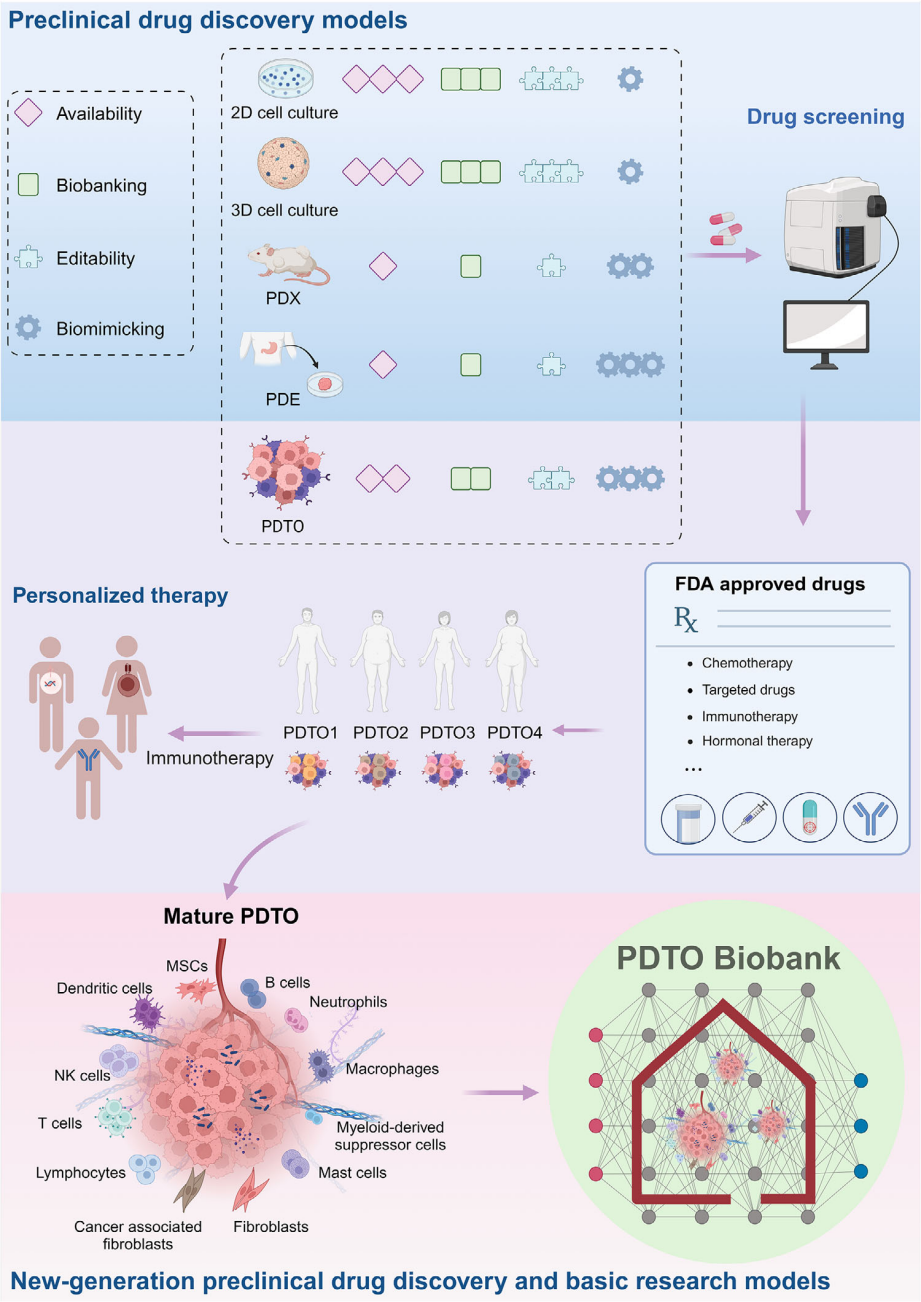
Future PDTOs are potent models for basic research and clinical uses. Due to its editability and biobanking capability, PDTOs are powerful in vitro models for personalized drug screening, new drug discovery, and precision medicine. Mature PDTOs as cancer avatars could replace animal models.

Furthermore, in addition to bacteria, tumours also harbour fungi and other microorganisms,^224^ which present new challenges for the PDTO model. However, it must be mentioned that compared to the PDE model, the PDTO model has more confidence in overcoming these difficulties appropriately (Figure 6). Based on its plasticity advantages, it can better introduce factors, including exosomes, ion elements and microorganisms. With the establishment of the PDTO biobank, systematic and complete models can be developed more freely and flexibly for different research modes to meet various research challenges.

## 6 PERSPECTIVES AND CONCLUSION

PDTOs and tumour assembloids represent groundbreaking and sophisticated models with significant advantages compared to the traditional models in cancer research. Nevertheless, the current PDTOs have limitations. For example, the facile PDTO construction approaches are not fully established. At the technical level, the main challenge is reappearing the IME for all types of cancers. It is crucial to consider renewable immune cell populations, angiogenesis, microorganisms, and a dynamic condition of nutrients to recapitulate the native TME. Another challenge is the construction of the PDTO biobank, which benefits the growing demands of basic research and clinical treatment. Researchers must address questions, including the limited availability of cell sources, the challenges of reassembling multiple cell types, time‐consuming and expensive culture methods, and the immature culture conditions for long‐term expansion and storage. Although PDTOs can better represent the genetic characteristics of the original tumours, they still cannot fully recapitulate the entire genetic landscape of native tumours.

Nevertheless, there is hope that improvements in sequencing accuracy, advancements in gene editing technology and the evolution of AI will gradually resolve the genetic limitations of PDTOs, allowing for the construction of more physiologically relevant models. As PDTO protocol and biobank become more successful, the safety and efficacy of pre‐clinical research are expected to be enhanced. Our review argues that the PDTOs for pre‐clinical immunotherapy screening will be the first step towards a broader research field. The success of PDTOs in immunotherapy replaces animals as a prime model for pre‐clinical studies and, more importantly, advances the research revolution from cells to organoids.

## AUTHOR CONTRIBUTIONS

J Mei and PY Wang conceived the manuscript; J Mei, XJ Liu, HX Tian, YX Chen, Y Cao and J Zeng searched for the papers and made the outline; J Mei, XJ Liu and HX Tian wrote the initial draft; J Mei, XJ Liu, HX Tian and YX Chen designed and drew the figures; J Mei, HX Tian, YC Liu and YP Chen checked all references and formatting; Y Gao, JY Yin and PY Wang supervised and edited the manuscript. J Mei, XJ Liu and HX Tian contributed equally to this work. All authors revised and contributed to the final version of the manuscript. Figures were created with biorender.com.

## CONFLICT OF INTEREST STATEMENT

The authors declare that there is no conflict of interest.

## ETHICS STATEMENT

Not applicable.
